# Dr. Emma Ardelia Buck Runnion

**Published:** 1913-06

**Authors:** 


Dr. Emma Ardelia Buck Runnion, Syracuse, 1888, a resident of Syracuse, died February 13, of lobar pneumonia, aged 58.

